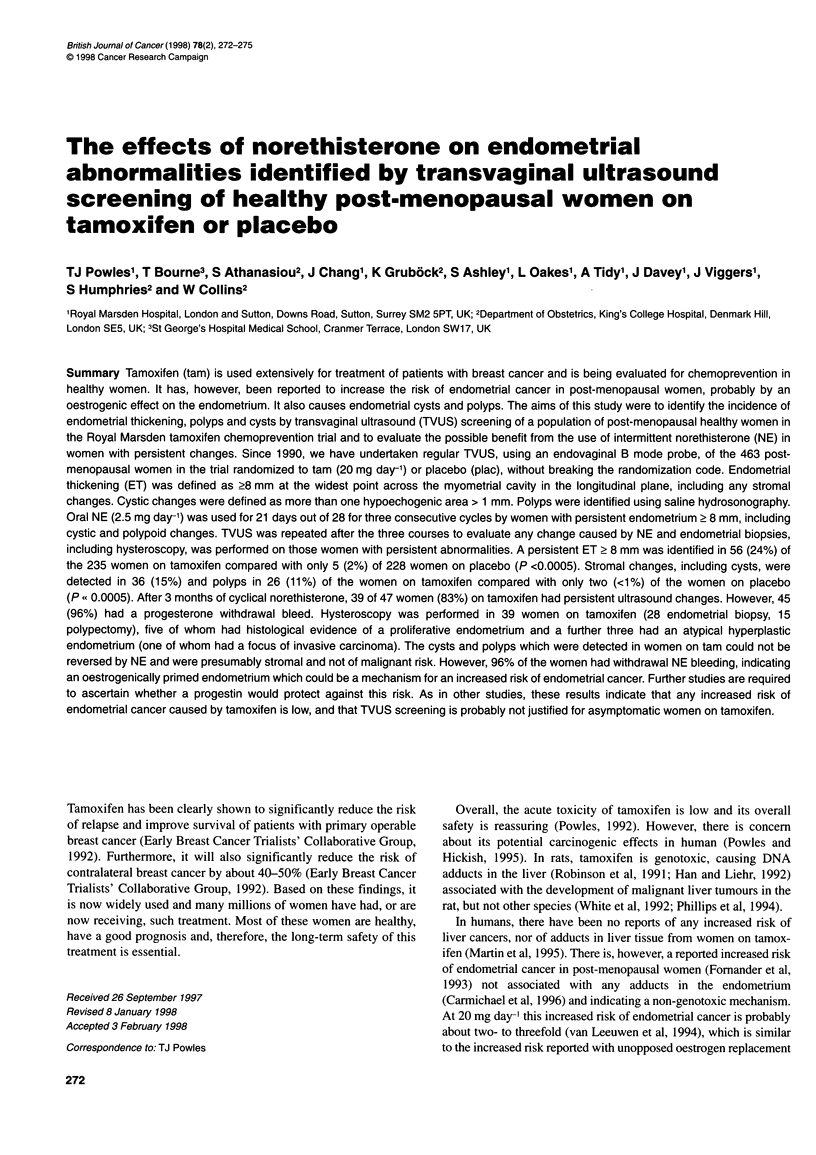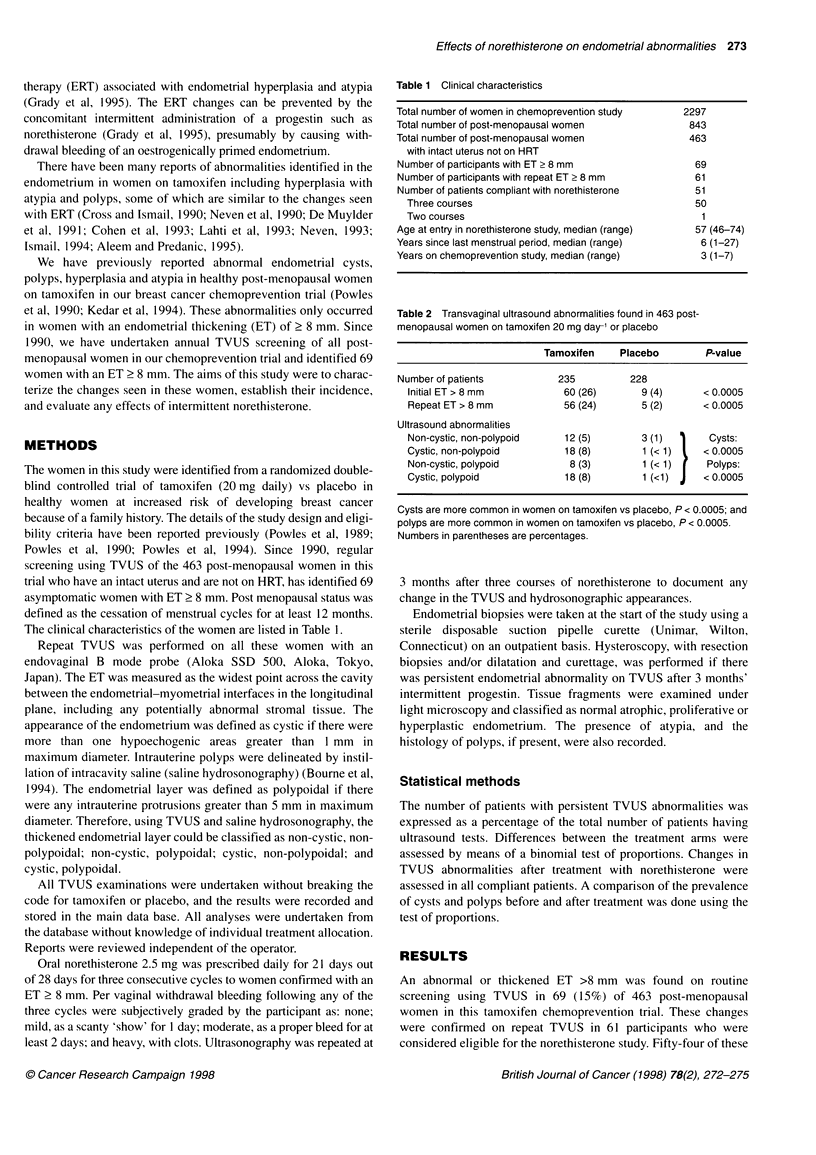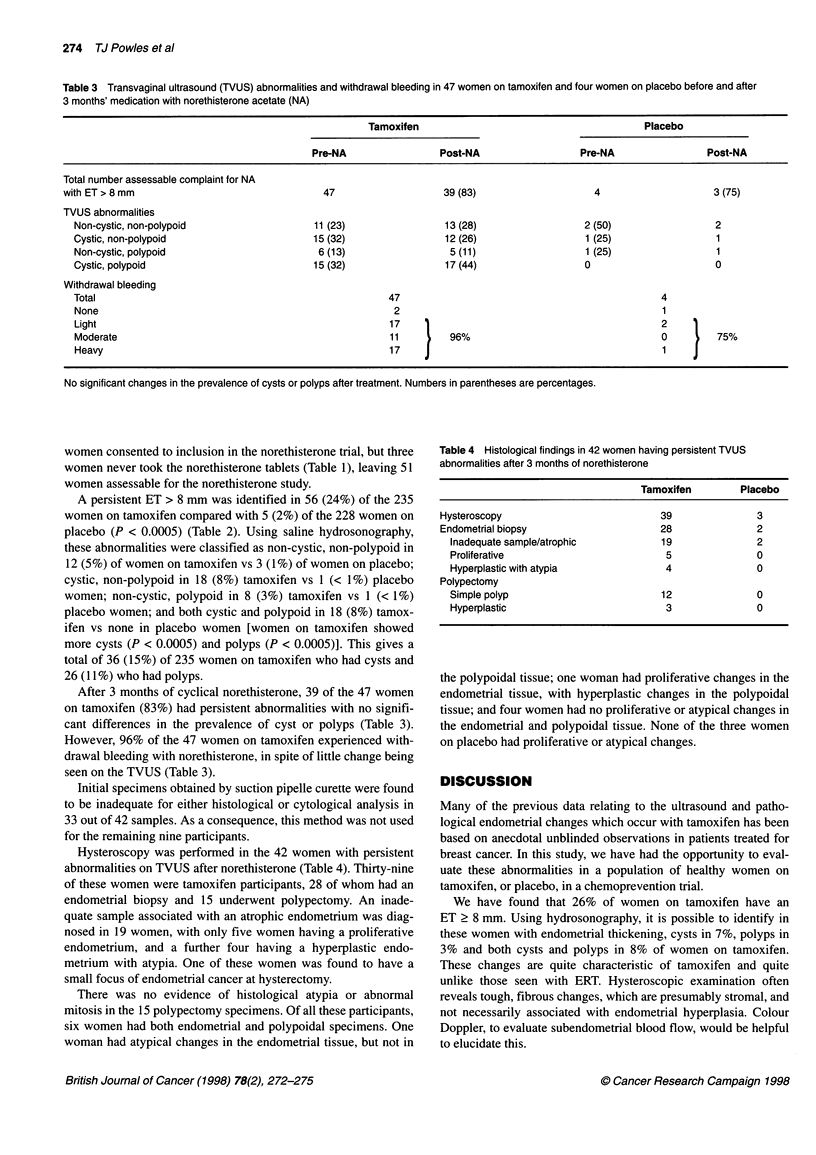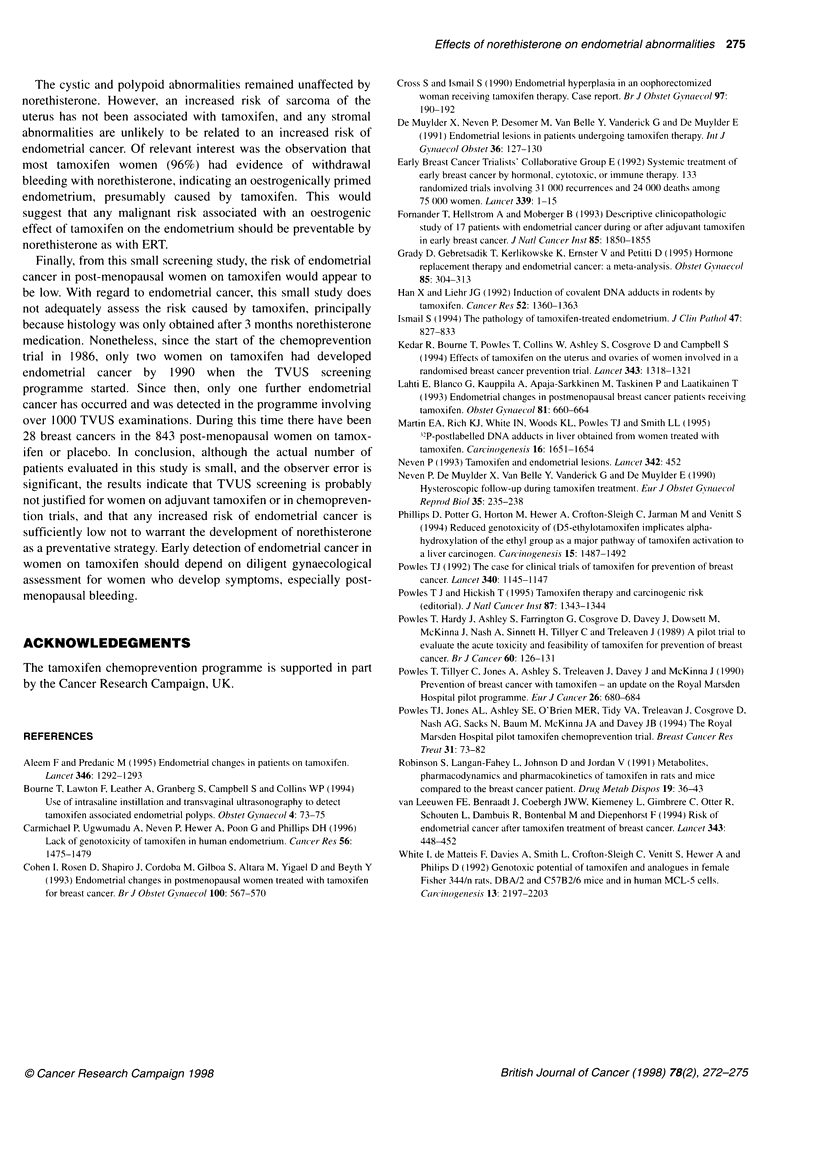# The effects of norethisterone on endometrial abnormalities identified by transvaginal ultrasound screening of healthy post-menopausal women on tamoxifen or placebo.

**DOI:** 10.1038/bjc.1998.477

**Published:** 1998-07

**Authors:** T. J. Powles, T. Bourne, S. Athanasiou, J. Chang, K. Gruböck, S. Ashley, L. Oakes, A. Tidy, J. Davey, J. Viggers, S. Humphries, W. Collins

**Affiliations:** Royal Marsden Hospital, London, UK.

## Abstract

Tamoxifen (tam) is used extensively for treatment of patients with breast cancer and is being evaluated for chemoprevention in healthy women. It has, however, been reported to increase the risk of endometrial cancer in post-menopausal women, probably by an oestrogenic effect on the endometrium. It also causes endometrial cysts and polyps. The aims of this study were to identify the incidence of endometrial thickening, polyps and cysts by transvaginal ultrasound (TVUS) screening of a population of post-menopausal healthy women in the Royal Marsden tamoxifen chemoprevention trial and to evaluate the possible benefit from the use of intermittent norethisterone (NE) in women with persistent changes. Since 1990, we have undertaken regular TVUS, using an endovaginal B mode probe, of the 463 post-menopausal women in the trial randomized to tam (20 mg day(-1)) or placebo (plac), without breaking the randomization code. Endometrial thickening (ET) was defined as > or = 8 mm at the widest point across the myometrial cavity in the longitudinal plane, including any stromal changes. Cystic changes were defined as more than one hypoechogenic area > 1 mm. Polyps were identified using saline hydrosonography. Oral NE (2.5 mg day(-1)) was used for 21 days out of 28 for three consecutive cycles by women with persistent endometrium > or = 8 mm, including cystic and polypoid changes. TVUS was repeated after the three courses to evaluate any change caused by NE and endometrial biopsies, including hysteroscopy, was performed on those women with persistent abnormalities. A persistent ET > or = 8 mm was identified in 56 (24%) of the 235 women on tamoxifen compared with only 5 (2%) of 228 women on placebo (P <0.0005). Stromal changes, including cysts, were detected in 36 (15%) and polyps in 26 (11%) of the women on tamoxifen compared with only two (< 1%) of the women on placebo (P << 0.0005). After 3 months of cyclical norethisterone, 39 of 47 women (83%) on tamoxifen had persistent ultrasound changes. However, 45 (96%) had a progesterone withdrawal bleed. Hysteroscopy was performed in 39 women on tamoxifen (28 endometrial biopsy, 15 polypectomy), five of whom had histological evidence of a proliferative endometrium and a further three had an atypical hyperplastic endometrium (one of whom had a focus of invasive carcinoma). The cysts and polyps which were detected in women on tam could not be reversed by NE and were presumably stromal and not of malignant risk. However, 96% of the women had withdrawal NE bleeding, indicating an oestrogenically primed endometrium which could be a mechanism for an increased risk of endometrial cancer. Further studies are required to ascertain whether a progestin would protect against this risk. As in other studies, these results indicate that any increased risk of endometrial cancer caused by tamoxifen is low, and that TVUS screening is probably not justified for asymptomatic women on tamoxifen.


British Journal of Cancer (1998) 78(2), 272–275
© 1998 Cancer Research Campaign

### The effects of norethisterone on endometrial abnormalities identified by transvaginal ultrasound screening of healthy post-menopausal women on tamoxifen or placebo

TJ Powles[1], T Bourne[3], S Athanasiou[2], J Chang[1], K Grubóck[2], S Ashley[1], L Oakes[1], A Tidy[1], J Davey[1], J Viggers[1], S Humphries[2] and W Collins[2]

[1]Royal Marsden Hospital, London and Sutton, Downs Road, Sutton, Surrey SM2 5PT, UK; [2]Department of Obstetrics, King's College Hospital, Denmark Hill, London SE5, UK; [3]St George's Hospital Medical School, Cranmer Terrace, London SW17, UK

**Summary** Tamoxifen (tam) is used extensively for treatment of patients with breast cancer and is being evaluated for chemoprevention in healthy women. It has, however, been reported to increase the risk of endometrial cancer in post-menopausal women, probably by an oestrogenic effect on the endometrium. It also causes endometrial cysts and polyps. The aims of this study were to identify the incidence of endometrial thickening, polyps and cysts by transvaginal ultrasound (TVUS) screening of a population of post-menopausal healthy women in the Royal Marsden tamoxifen chemoprevention trial and to evaluate the possible benefit from the use of intermittent norethisterone (NE) in women with persistent changes. Since 1990, we have undertaken regular TVUS, using an endovaginal B mode probe, of the 463 post-menopausal women in the trial randomized to tam (20 mg day$^{-1}$) or placebo (plac), without breaking the randomization code. Endometrial thickening (ET) was defined as ≥8 mm at the widest point across the myometrial cavity in the longitudinal plane, including any stromal changes. Cystic changes were defined as more than one hypoechogenic area > 1 mm. Polyps were identified using saline hydrosonography. Oral NE (2.5 mg day$^{-1}$) was used for 21 days out of 28 for three consecutive cycles by women with persistent endometrium ≥ 8 mm, including cystic and polypoid changes. TVUS was repeated after the three courses to evaluate any change caused by NE and endometrial biopsies, including hysteroscopy, was performed on those women with persistent abnormalities. A persistent ET ≥ 8 mm was identified in 56 (24%) of the 235 women on tamoxifen compared with only 5 (2%) of 228 women on placebo ($P$ <0.0005). Stromal changes, including cysts, were detected in 36 (15%) and polyps in 26 (11%) of the women on tamoxifen compared with only two (<1%) of the women on placebo ($P$ « 0.0005). After 3 months of cyclical norethisterone, 39 of 47 women (83%) on tamoxifen had persistent ultrasound changes. However, 45 (96%) had a progesterone withdrawal bleed. Hysteroscopy was performed in 39 women on tamoxifen (28 endometrial biopsy, 15 polypectomy), five of whom had histological evidence of a proliferative endometrium and a further three had an atypical hyperplastic endometrium (one of whom had a focus of invasive carcinoma). The cysts and polyps which were detected in women on tam could not be reversed by NE and were presumably stromal and not of malignant risk. However, 96% of the women had withdrawal NE bleeding, indicating an oestrogenically primed endometrium which could be a mechanism for an increased risk of endometrial cancer. Further studies are required to ascertain whether a progestin would protect against this risk. As in other studies, these results indicate that any increased risk of endometrial cancer caused by tamoxifen is low, and that TVUS screening is probably not justified for asymptomatic women on tamoxifen.

Tamoxifen has been clearly shown to significantly reduce the risk of relapse and improve survival of patients with primary operable breast cancer (Early Breast Cancer Trialists' Collaborative Group, 1992). Furthermore, it will also significantly reduce the risk of contralateral breast cancer by about 40–50% (Early Breast Cancer Trialists' Collaborative Group, 1992). Based on these findings, it is now widely used and many millions of women have had, or are now receiving, such treatment. Most of these women are healthy, have a good prognosis and, therefore, the long-term safety of this treatment is essential.

*Received 26 September 1997*
*Revised 8 January 1998*
*Accepted 3 February 1998*

*Correspondence to:* TJ Powles

Overall, the acute toxicity of tamoxifen is low and its overall safety is reassuring (Powles, 1992). However, there is concern about its potential carcinogenic effects in human (Powles and Hickish, 1995). In rats, tamoxifen is genotoxic, causing DNA adducts in the liver (Robinson et al, 1991; Han and Liehr, 1992) associated with the development of malignant liver tumours in the rat, but not other species (White et al, 1992; Phillips et al, 1994).

In humans, there have been no reports of any increased risk of liver cancers, nor of adducts in liver tissue from women on tamoxifen (Martin et al, 1995). There is, however, a reported increased risk of endometrial cancer in post-menopausal women (Fornander et al, 1993) not associated with any adducts in the endometrium (Carmichael et al, 1996) and indicating a non-genotoxic mechanism. At 20 mg day$^{-1}$ this increased risk of endometrial cancer is probably about two- to threefold (van Leeuwen et al, 1994), which is similar to the increased risk reported with unopposed oestrogen replacement therapy (ERT) associated with endometrial hyperplasia and atypia (Grady et al, 1995). The ERT changes can be prevented by the concomitant intermittent administration of a progestin such as norethisterone (Grady et al, 1995), presumably by causing withdrawal bleeding of an oestrogenically primed endometrium.

There have been many reports of abnormalities identified in the endometrium in women on tamoxifen including hyperplasia with atypia and polyps, some of which are similar to the changes seen with ERT (Cross and Ismail, 1990; Neven et al, 1990; De Muylder et al, 1991; Cohen et al, 1993; Lahti et al, 1993; Neven, 1993; Ismail, 1994; Aleem and Predanic, 1995).

We have previously reported abnormal endometrial cysts, polyps, hyperplasia and atypia in healthy post-menopausal women on tamoxifen in our breast cancer chemoprevention trial (Powles et al, 1990; Kedar et al, 1994). These abnormalities only occurred in women with an endometrial thickening (ET) of ≥ 8 mm. Since 1990, we have undertaken annual TVUS screening of all post-menopausal women in our chemoprevention trial and identified 69 women with an ET ≥ 8 mm. The aims of this study were to characterize the changes seen in these women, establish their incidence, and evaluate any effects of intermittent norethisterone.

### METHODS

The women in this study were identified from a randomized double-blind controlled trial of tamoxifen (20 mg daily) vs placebo in healthy women at increased risk of developing breast cancer because of a family history. The details of the study design and eligibility criteria have been reported previously (Powles et al, 1989; Powles et al, 1990; Powles et al, 1994). Since 1990, regular screening using TVUS of the 463 post-menopausal women in this trial who have an intact uterus and are not on HRT, has identified 69 asymptomatic women with ET ≥ 8 mm. Post menopausal status was defined as the cessation of menstrual cycles for at least 12 months. The clinical characteristics of the women are listed in Table 1.

Repeat TVUS was performed on all these women with an endovaginal B mode probe (Aloka SSD 500, Aloka, Tokyo, Japan). The ET was measured as the widest point across the cavity between the endometrial–myometrial interfaces in the longitudinal plane, including any potentially abnormal stromal tissue. The appearance of the endometrium was defined as cystic if there were more than one hypoechogenic areas greater than 1 mm in maximum diameter. Intrauterine polyps were delineated by instillation of intracavity saline (saline hydrosonography) (Bourne et al, 1994). The endometrial layer was defined as polypoidal if there were any intrauterine protrusions greater than 5 mm in maximum diameter. Therefore, using TVUS and saline hydrosonography, the thickened endometrial layer could be classified as non-cystic, non-polypoidal; non-cystic, polypoidal; cystic, non-polypoidal; and cystic, polypoidal.

All TVUS examinations were undertaken without breaking the code for tamoxifen or placebo, and the results were recorded and stored in the main data base. All analyses were undertaken from the database without knowledge of individual treatment allocation. Reports were reviewed independent of the operator.

Oral norethisterone 2.5 mg was prescribed daily for 21 days out of 28 days for three consecutive cycles to women confirmed with an ET ≥ 8 mm. Per vaginal withdrawal bleeding following any of the three cycles were subjectively graded by the participant as: none; mild, as a scanty 'show' for 1 day; moderate, as a proper bleed for at least 2 days; and heavy, with clots. Ultrasonography was repeated at 3 months after three courses of norethisterone to document any change in the TVUS and hydrosonographic appearances.

**Table 1** Clinical characteristics

| | |
|---|---|
| Total number of women in chemoprevention study | 2297 |
| Total number of post-menopausal women | 843 |
| Total number of post-menopausal women with intact uterus not on HRT | 463 |
| Number of participants with ET ≥ 8 mm | 69 |
| Number of participants with repeat ET ≥ 8 mm | 61 |
| Number of patients compliant with norethisterone | 51 |
| Three courses | 50 |
| Two courses | 1 |
| Age at entry in norethisterone study, median (range) | 57 (46–74) |
| Years since last menstrual period, median (range) | 6 (1–27) |
| Years on chemoprevention study, median (range) | 3 (1–7) |

**Table 2** Transvaginal ultrasound abnormalities found in 463 post-menopausal women on tamoxifen 20 mg day$^{-1}$ or placebo

| | Tamoxifen | Placebo | *P*-value |
|---|---|---|---|
| Number of patients | 235 | 228 | |
| Initial ET > 8 mm | 60 (26) | 9 (4) | < 0.0005 |
| Repeat ET > 8 mm | 56 (24) | 5 (2) | < 0.0005 |
| Ultrasound abnormalities | | | |
| Non-cystic, non-polypoid | 12 (5) | 3 (1) | Cysts: |
| Cystic, non-polypoid | 18 (8) | 1 (< 1) | < 0.0005 |
| Non-cystic, polypoid | 8 (3) | 1 (< 1) | Polyps: |
| Cystic, polypoid | 18 (8) | 1 (<1) | < 0.0005 |

Cysts are more common in women on tamoxifen vs placebo, *P* < 0.0005; and polyps are more common in women on tamoxifen vs placebo, *P* < 0.0005. Numbers in parentheses are percentages.

Endometrial biopsies were taken at the start of the study using a sterile disposable suction pipelle curette (Unimar, Wilton, Connecticut) on an outpatient basis. Hysteroscopy, with resection biopsies and/or dilatation and curettage, was performed if there was persistent endometrial abnormality on TVUS after 3 months' intermittent progestin. Tissue fragments were examined under light microscopy and classified as normal atrophic, proliferative or hyperplastic endometrium. The presence of atypia, and the histology of polyps, if present, were also recorded.

### Statistical methods

The number of patients with persistent TVUS abnormalities was expressed as a percentage of the total number of patients having ultrasound tests. Differences between the treatment arms were assessed by means of a binomial test of proportions. Changes in TVUS abnormalities after treatment with norethisterone were assessed in all compliant patients. A comparison of the prevalence of cysts and polyps before and after treatment was done using the test of proportions.

### RESULTS

An abnormal or thickened ET >8 mm was found on routine screening using TVUS in 69 (15%) of 463 post-menopausal women in this tamoxifen chemoprevention trial. These changes were confirmed on repeat TVUS in 61 participants who were considered eligible for the norethisterone study. Fifty-four of these

© Cancer Research Campaign 1998

**Table 3** Transvaginal ultrasound (TVUS) abnormalities and withdrawal bleeding in 47 women on tamoxifen and four women on placebo before and after 3 months' medication with norethisterone acetate (NA)

| | Tamoxifen | | | Placebo | | |
|---|---|---|---|---|---|---|
| | Pre-NA | | Post-NA | Pre-NA | | Post-NA |
| Total number assessable complaint for NA with ET > 8 mm | 47 | | 39 (83) | 4 | | 3 (75) |
| TVUS abnormalities | | | | | | |
| Non-cystic, non-polypoid | 11 (23) | | 13 (28) | 2 (50) | | 2 |
| Cystic, non-polypoid | 15 (32) | | 12 (26) | 1 (25) | | 1 |
| Non-cystic, polypoid | 6 (13) | | 5 (11) | 1 (25) | | 1 |
| Cystic, polypoid | 15 (32) | | 17 (44) | 0 | | 0 |
| Withdrawal bleeding | | | | | | |
| Total | | 47 | | | 4 | |
| None | | 2 | | | 1 | |
| Light | | 17 | 96% | | 2 | 75% |
| Moderate | | 11 | | | 0 | |
| Heavy | | 17 | | | 1 | |

No significant changes in the prevalence of cysts or polyps after treatment. Numbers in parentheses are percentages.

women consented to inclusion in the norethisterone trial, but three women never took the norethisterone tablets (Table 1), leaving 51 women assessable for the norethisterone study.

A persistent ET > 8 mm was identified in 56 (24%) of the 235 women on tamoxifen compared with 5 (2%) of the 228 women on placebo ($P < 0.0005$) (Table 2). Using saline hydrosonography, these abnormalities were classified as non-cystic, non-polypoid in 12 (5%) of women on tamoxifen vs 3 (1%) of women on placebo; cystic, non-polypoid in 18 (8%) tamoxifen vs 1 (< 1%) placebo women; non-cystic, polypoid in 8 (3%) tamoxifen vs 1 (< 1%) placebo women; and both cystic and polypoid in 18 (8%) tamoxifen vs none in placebo women [women on tamoxifen showed more cysts ($P < 0.0005$) and polyps ($P < 0.0005$)]. This gives a total of 36 (15%) of 235 women on tamoxifen who had cysts and 26 (11%) who had polyps.

After 3 months of cyclical norethisterone, 39 of the 47 women on tamoxifen (83%) had persistent abnormalities with no significant differences in the prevalence of cyst or polyps (Table 3). However, 96% of the 47 women on tamoxifen experienced withdrawal bleeding with norethisterone, in spite of little change being seen on the TVUS (Table 3).

Initial specimens obtained by suction pipelle curette were found to be inadequate for either histological or cytological analysis in 33 out of 42 samples. As a consequence, this method was not used for the remaining nine participants.

Hysteroscopy was performed in the 42 women with persistent abnormalities on TVUS after norethisterone (Table 4). Thirty-nine of these women were tamoxifen participants, 28 of whom had an endometrial biopsy and 15 underwent polypectomy. An inadequate sample associated with an atrophic endometrium was diagnosed in 19 women, with only five women having a proliferative endometrium, and a further four having a hyperplastic endometrium with atypia. One of these women was found to have a small focus of endometrial cancer at hysterectomy.

There was no evidence of histological atypia or abnormal mitosis in the 15 polypectomy specimens. Of all these participants, six women had both endometrial and polypoidal specimens. One woman had atypical changes in the endometrial tissue, but not in the polypoidal tissue; one woman had proliferative changes in the endometrial tissue, with hyperplastic changes in the polypoidal tissue; and four women had no proliferative or atypical changes in the endometrial and polypoidal tissue. None of the three women on placebo had proliferative or atypical changes.

**Table 4** Histological findings in 42 women having persistent TVUS abnormalities after 3 months of norethisterone

| | Tamoxifen | Placebo |
|---|---|---|
| Hysteroscopy | 39 | 3 |
| Endometrial biopsy | 28 | 2 |
| Inadequate sample/atrophic | 19 | 2 |
| Proliferative | 5 | 0 |
| Hyperplastic with atypia | 4 | 0 |
| Polypectomy | | |
| Simple polyp | 12 | 0 |
| Hyperplastic | 3 | 0 |

### DISCUSSION

Many of the previous data relating to the ultrasound and pathological endometrial changes which occur with tamoxifen has been based on anecdotal unblinded observations in patients treated for breast cancer. In this study, we have had the opportunity to evaluate these abnormalities in a population of healthy women on tamoxifen, or placebo, in a chemoprevention trial.

We have found that 26% of women on tamoxifen have an ET ≥ 8 mm. Using hydrosonography, it is possible to identify in these women with endometrial thickening, cysts in 7%, polyps in 3% and both cysts and polyps in 8% of women on tamoxifen. These changes are quite characteristic of tamoxifen and quite unlike those seen with ERT. Hysteroscopic examination often reveals tough, fibrous changes, which are presumably stromal, and not necessarily associated with endometrial hyperplasia. Colour Doppler, to evaluate subendometrial blood flow, would be helpful to elucidate this.

 © Cancer Research Campaign 1998

The cystic and polypoid abnormalities remained unaffected by norethisterone. However, an increased risk of sarcoma of the uterus has not been associated with tamoxifen, and any stromal abnormalities are unlikely to be related to an increased risk of endometrial cancer. Of relevant interest was the observation that most tamoxifen women (96%) had evidence of withdrawal bleeding with norethisterone, indicating an oestrogenically primed endometrium, presumably caused by tamoxifen. This would suggest that any malignant risk associated with an oestrogenic effect of tamoxifen on the endometrium should be preventable by norethisterone as with ERT.

Finally, from this small screening study, the risk of endometrial cancer in post-menopausal women on tamoxifen would appear to be low. With regard to endometrial cancer, this small study does not adequately assess the risk caused by tamoxifen, principally because histology was only obtained after 3 months norethisterone medication. Nonetheless, since the start of the chemoprevention trial in 1986, only two women on tamoxifen had developed endometrial cancer by 1990 when the TVUS screening programme started. Since then, only one further endometrial cancer has occurred and was detected in the programme involving over 1000 TVUS examinations. During this time there have been 28 breast cancers in the 843 post-menopausal women on tamoxifen or placebo. In conclusion, although the actual number of patients evaluated in this study is small, and the observer error is significant, the results indicate that TVUS screening is probably not justified for women on adjuvant tamoxifen or in chemoprevention trials, and that any increased risk of endometrial cancer is sufficiently low not to warrant the development of norethisterone as a preventative strategy. Early detection of endometrial cancer in women on tamoxifen should depend on diligent gynaecological assessment for women who develop symptoms, especially post-menopausal bleeding.

### ACKNOWLEDGEMENTS

The tamoxifen chemoprevention programme is supported in part by the Cancer Research Campaign, UK.

### REFERENCES

Aleem F and Predanic M (1995) Endometrial changes in patients on tamoxifen. *Lancet* **346**: 1292–1293

Bourne T, Lawton F, Leather A, Granberg S, Campbell S and Collins WP (1994) Use of intrasaline instillation and transvaginal ultrasonography to detect tamoxifen associated endometrial polyps. *Obstet Gynaecol* **4**: 73–75

Carmichael P, Ugwumadu A, Neven P, Hewer A, Poon G and Phillips DH (1996) Lack of genotoxicity of tamoxifen in human endometrium. *Cancer Res* **56**: 1475–1479

Cohen I, Rosen D, Shapiro J, Cordoba M, Gilboa S, Altara M, Yigael D and Beyth Y (1993) Endometrial changes in postmenopausal women treated with tamoxifen for breast cancer. *Br J Obstet Gynaecol* **100**: 567–570

Cross S and Ismail S (1990) Endometrial hyperplasia in an oophorectomized woman receiving tamoxifen therapy. Case report. *Br J Obstet Gynaecol* **97**: 190–192

De Muylder X, Neven P, Desomer M, Van Belle Y, Vanderick G and De Muylder E (1991) Endometrial lesions in patients undergoing tamoxifen therapy. *Int J Gynaecol Obstet* **36**: 127–130

Early Breast Cancer Trialists' Collaborative Group E (1992) Systemic treatment of early breast cancer by hormonal, cytotoxic, or immune therapy. 133 randomized trials involving 31 000 recurrences and 24 000 deaths among 75 000 women. *Lancet* **339**: 1–15

Fornander T, Hellstrom A and Moberger B (1993) Descriptive clinicopathologic study of 17 patients with endometrial cancer during or after adjuvant tamoxifen in early breast cancer. *J Natl Cancer Inst* **85**: 1850–1855

Grady D, Gebretsadik T, Kerlikowske K, Ernster V and Petitti D (1995) Hormone replacement therapy and endometrial cancer: a meta-analysis. *Obstet Gynaecol* **85**: 304–313

Han X and Liehr JG (1992) Induction of covalent DNA adducts in rodents by tamoxifen. *Cancer Res* **52**: 1360–1363

Ismail S (1994) The pathology of tamoxifen-treated endometrium. *J Clin Pathol* **47**: 827–833

Kedar R, Bourne T, Powles T, Collins W, Ashley S, Cosgrove D and Campbell S (1994) Effects of tamoxifen on the uterus and ovaries of women involved in a randomised breast cancer prevention trial. *Lancet* **343**: 1318–1321

Lahti E, Blanco G, Kauppila A, Apaja-Sarkkinen M, Taskinen P and Laatikainen T (1993) Endometrial changes in postmenopausal breast cancer patients receiving tamoxifen. *Obstet Gynaecol* **81**: 660–664

Martin EA, Rich KJ, White IN, Woods KL, Powles TJ and Smith LL (1995) $^{32}$P-postlabelled DNA adducts in liver obtained from women treated with tamoxifen. *Carcinogenesis* **16**: 1651–1654

Neven P (1993) Tamoxifen and endometrial lesions. *Lancet* **342**: 452

Neven P, De Muylder X, Van Belle Y, Vanderick G and De Muylder E (1990) Hysteroscopic follow-up during tamoxifen treatment. *Eur J Obstet Gynaecol Reprod Biol* **35**: 235–238

Phillips D, Potter G, Horton M, Hewer A, Crofton-Sleigh C, Jarman M and Venitt S (1994) Reduced genotoxicity of (D5-ethylotamoxifen implicates alpha-hydroxylation of the ethyl group as a major pathway of tamoxifen activation to a liver carcinogen. *Carcinogenesis* **15**: 1487–1492

Powles TJ (1992) The case for clinical trials of tamoxifen for prevention of breast cancer. *Lancet* **340**: 1145–1147

Powles T J and Hickish T (1995) Tamoxifen therapy and carcinogenic risk (editorial). *J Natl Cancer Inst* **87**: 1343–1344

Powles T, Hardy J, Ashley S, Farrington G, Cosgrove D, Davey J, Dowsett M, McKinna J, Nash A, Sinnett H, Tillyer C and Treleaven J (1989) A pilot trial to evaluate the acute toxicity and feasibility of tamoxifen for prevention of breast cancer. *Br J Cancer* **60**: 126–131

Powles T, Tillyer C, Jones A, Ashley S, Treleaven J, Davey J and McKinna J (1990) Prevention of breast cancer with tamoxifen – an update on the Royal Marsden Hospital pilot programme. *Eur J Cancer* **26**: 680–684

Powles TJ, Jones AL, Ashley SE, O'Brien MER, Tidy VA, Treleavan J, Cosgrove D, Nash AG, Sacks N, Baum M, McKinna JA and Davey JB (1994) The Royal Marsden Hospital pilot tamoxifen chemoprevention trial. *Breast Cancer Res Treat* **31**: 73–82

Robinson S, Langan-Fahey L, Johnson D and Jordan V (1991) Metabolites, pharmacodynamics and pharmacokinetics of tamoxifen in rats and mice compared to the breast cancer patient. *Drug Metab Dispos* **19**: 36–43

van Leeuwen FE, Benraadt J, Coebergh JWW, Kiemeney L, Gimbrere C, Otter R, Schouten L, Dambuis R, Bontenbal M and Diepenhorst F (1994) Risk of endometrial cancer after tamoxifen treatment of breast cancer. *Lancet* **343**: 448–452

White I, de Matteis F, Davies A, Smith L, Crofton-Sleigh C, Venitt S, Hewer A and Philips D (1992) Genotoxic potential of tamoxifen and analogues in female Fisher 344/n rats, DBA/2 and C57B2/6 mice and in human MCL-5 cells. *Carcinogenesis* **13**: 2197–2203

© Cancer Research Campaign 1998